# BioCIDER: a Contextualisation InDEx for biological Resources discovery

**DOI:** 10.1093/bioinformatics/btx213

**Published:** 2017-04-12

**Authors:** Carlos Horro, Martin Cook, Teresa K Attwood, Michelle D Brazas, John M Hancock, Patricia Palagi, Manuel Corpas, Rafael Jimenez

**Affiliations:** 1 Elixir Department, Earlham Institute, Norwich Research Park Innovation Centre, Norwich, UK; 2 ELIXIR Hub, The European Bioinformatics Institute (EMBL-EBI), Wellcome Trust Genome Campus, Hinxton, UK; 3 School of Computer Science, The University of Manchester, Manchester, UK; 4 Informatics and Bio-computing, Ontario Institute for Cancer Research, Toronto, Canada; 5 SIB Training Group, SIB Swiss Institute of Bioinformatics, Lausanne, Switzerland; 6 Repositive, Future Business Centre, Kings' Hedges Road, Cambridge, UK

## Abstract

**Summary:**

The vast, uncoordinated proliferation of bioinformatics resources (databases, software tools, training materials etc.) makes it difficult for users to find them. To facilitate their discovery, various services are being developed to collect such resources into registries. We have developed BioCIDER, which, rather like online shopping ‘recommendations’, provides a contextualization index to help identify biological resources relevant to the content of the sites in which it is embedded.

**Availability and Implementation:**

BioCIDER (www.biocider.org) is an open-source platform. Documentation is available online (https://goo.gl/Klc51G), and source code is freely available via GitHub (https://github.com/BioCIDER). The BioJS widget that enables websites to embed contextualization is available from the BioJS registry (http://biojs.io/). All code is released under an MIT licence.

## 1 Introduction

Life-science resources (i.e. databases, tools, training materials, courses and event information) are many, diverse, widely dispersed and hard to find. The 2016 Nucleic Acids Research (NAR) Database Issue (Ridgen *et al.*, 2016) reported 1685 major databases in the molecular biology domain, while the latest NAR Web Server Issue (Editorial: Nucleic Acids Research annual Web Server Issue in 2016, 2016) presented 94 new resources for 2016 alone. It is thus difficult for researchers either to be aware of or to be familiar with all current and relevant research assets, compromising their uptake and general utility. Researchers do not just need better but, crucially, more practical ways to discover resources. Discoverability can be significantly enhanced if resources are exposed to users in context with the information they are currently browsing; if sufficiently relevant and well placed, this strategy may introduce advantageous new information and obviate the need to browse further. An analogy can be drawn, e.g. with prominent online retailers that use widgets to display ‘*customers also bought*’ or ‘*recommended items based on your search*’. To our knowledge, there is no life science-focused service that provides contextualized information driving researchers to discover relevant databases, tools, events and training materials. To address this gap, we have developed BioCIDER, a *Contextualization InDEx for biological Resource discovery*. BioCIDER automatically collects information (metadata and source description) from a variety of centralized registries, including the GOBLET training portal (Corpas *et al.*, 2015), the Bio.tools service registry (Ison *et al.*, 2015), the iAnn collaborative event dissemination portal (Jimenez *et al.*, 2013) and TeSS, the ELIXIR training portal (https://tess.elixir-uk.org/); others (e.g. biosharing.org; McQuilton *et al*, 2016) will be added in future. BioCIDER can be embedded in any website via its companion widget from the BioJavaScript (BioJS) open source library of components (Corpas *et al.*, 2014).

## 2 Materials and methods

The BioCIDER service comprises three parts: (i) a set of Python scripts that periodically import data from different sources across the Internet (the so-called data-import layer); (ii) a centralized Solr index, which stores all the information collected by these scripts; and (iii) a Web service provided by the Solr indexing system (http://lucene.apache.org/solr/) that allows access to the data from any location (i.e. not necessarily through one specific client). The data-import layer is highly modularized, and allows addition of new scripts in order to incorporate additional data sources to the platform. Data from each source are updated independently and automatically, triggering specific procedures with different frequencies set by timers taking into account the known update frequencies of each site. Solr is an open-source search platform which features indexed text storage, allowing data from the import layer to be stored and sorted, making it possible to perform complex searches throughout its entire content rapidly. These searches can be done (i) locally, (ii) through a Web-management application or (iii) via a Web service whose URL is publicly available and is used by the BioCIDER widget. Once a query is sent to the Web service, a simple JSON-formatted (JavaScript Object Notation) file is retrieved.

The contextualization process is based on the Solr Term Frequency (TF)–Inverse Document Frequency (IDF) algorithm. This allows retrieval of lists of resources ordered by their similarity with the search phrase by measuring the TF (the number of times each term occurs in each document) and IDF (a measure of how common or rare the term is across all documents).

**Fig. 1:**
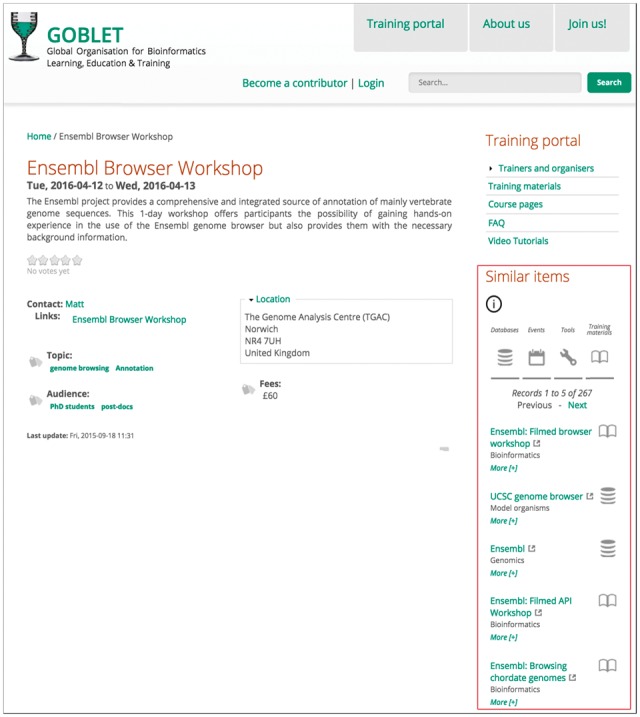
Screen-shot of the ‘*Ensembl Browser Workshop: Plants and Microbes*’ course page accessed from the GOBLET portal (www.mygoblet.org). The BioCIDER widget, framed inside the red rectangle, shows related databases, events, tools and training materials, contextualized to NGS. The widget is populated with short descriptions and links to relevant content on the course page. Original sources for each BioCIDER result can be accessed by clicking on them. The widget dynamically adapts to the shape and visual styles of its container, appearing as an integral part of the website

BioCIDER can be used in any website with bioinformatics content, and can be shared and re-used by the BioJS community (Yachdav *et al.*, 2015). As input, the BioCIDER widget requires a query phrase, and returns a list of results (red rectangle, Fig. 1) showing the names of known resources, with links to their original source for further information (the number and type of results shown is configurable). The more descriptive the input words, the more relevant the suggestions. The widget can be configured to retrieve input automatically from content displayed in the webpage being browsed; its functionality is easy to integrate—it works autonomously, without interfering with the website’s behaviour, and can be themed to match the design of the host site.

## 3 Conclusions

BioCIDER provides an infrastructure for intuitive, fast and non-intrusive discovery of bioinformatics databases, tools, training materials and events. Its Web service can be freely used by any client website or user, retrieving contextualized resource information in a simple JSON formatted file. This Web service is based on the Solr index system and its TF–IDF algorithm, which receives the query phrase from the client, measures the relevance to known resources, and returns a sorted, relevance-ranked list. An open source BioJS widget is provided to embed the query results in the host webpages. The BioCIDER widget is already being used by organizations such as GOBLET (Attwood *et al.*, 2015) and ELIXIR-UK (http://www.elixir-uk.org). Thus, users interested on NGS courses who have found the ‘*Introduction to NGS Bioinformatics’* course on the GOBLET Training Portal (http://www.mygoblet.org/training-portal/courses/introduction-ngs-bioinformatics) will also discover in the BioCIDER widget (called here ‘*Similar items*’) many topic-related training materials and events potentially useful to them.
